# Spatial clusters of risk and cartography of care for drug-resistant tuberculosis

**DOI:** 10.11606/s1518-8787.2025059006489

**Published:** 2025-06-11

**Authors:** Jaqueline Garcia de Almeida Ballestero, José Nildo de Barros Silva, Luiz Henrique Arroyo, Daniele Maria Pelissari, Isabela Zaccaro Rigolin, Pedro Fredemir Palha, Aline Aparecida Monroe, Quézia Rosa Ferreira, Gilberto da Cruz Leal, Leonora de Oliveira Teixeira, Yury Bitencourt da Costa, Ione Carvalho Pinto, Rubia Laine de Paula Andrade, Ricardo Alexandre Arcêncio

**Affiliations:** I Universidade de São Paulo Escola de Enfermagem de Ribeirão Preto Ribeirão Preto SP Brasil Universidade de São Paulo. Escola de Enfermagem de Ribeirão Preto. Ribeirão Preto, SP, Brasil; II Universidade de São Paulo Faculdade de Saúde Pública São Paulo SP Brasil Universidade de São Paulo. Faculdade de Saúde Pública. São Paulo, SP, Brasil

**Keywords:** Tuberculosis, Multidrug-resistant tuberculosis, Geographic mapping, Health Systems, Health Information Systems, Public Health Nursing

## Abstract

**OBJECTIVE:**

To identify spatial clusters of risk and map the care network for people with drug-resistant tuberculosis in the state of São Paulo.

**METHODS:**

This is an ecological study, carried out by collecting data from the Special Tuberculosis Treatment Information System (Site-TB) of people treated for drug-resistant tuberculosis from 2013 to 2020, in the state of São Paulo. Mapping was carried out using Kernel and scan statistic techniques.

**RESULTS:**

1,084 cases were reported in the period analyzed. São Paulo, Ribeirão Preto, Santos, Guarulhos, and Campinas were the municipalities with the highest number of cases. The spatial pattern of agglomeration of cases and referral centers for treatment were similar, with gaps in coverage in the southwest and northwest of the state. Six spatial clusters were identified: four low-risk and two high-risk, located in São Paulo, Diadema, Santos, and Guarujá.

**CONCLUSIONS:**

The concentration of cases and tertiary referral centers in metropolitan areas highlights inequalities in access to treatment for drug-resistant tuberculosis. These findings indicate the need for health policies to expand diagnosis and treatment, improving the control of drug-resistant tuberculosis in the state of São Paulo.

## INTRODUCTION

Drug-resistant tuberculosis consists of drug resistance to one or more standardized drugs for the treatment of tuberculosis^1^. Its most serious forms include multidrug-resistant tuberculosis and extensively drug-resistant tuberculosis, both of which have a high likelihood of having a negative impact on the lives of people with the disease. Resistance can be detected using the molecular rapid molecular (MRT-TB) in cases that are resistant to rifampicin^2-3^.

The challenges related to the diagnosis of resistant forms are complex, encompassing microbiological, clinical, social, organizational, and political aspects^4^. In 2022, of the 410,000 cases of multidrug-resistant or rifampicin-resistant tuberculosis estimated by the World Health Organization (WHO), only 175,650 (42.8%) were diagnosed and started treatment^4^. In Brazil, 1,104 cases of drug-resistant tuberculosis were reported in the same year, which is equivalent to around 67.1% of the total estimated by the WHO for the country^1^. In addition, the state of São Paulo had the second highest proportion of drug-resistant tuberculosis cases, with the municipality of São Paulo being the third in number of cases in the Brazilian context, from 2019 to 2023^5^.

Thus, although Brazil is not included in the list of the 30 countries with the highest burden of drug-resistant tuberculosis, the epidemiological profile of tuberculosis in the country has led the WHO to consider the country to be at high risk for the spread of resistant forms^6^, which represents a considerable public health issue.

When it comes to organizing care for drug-resistant tuberculosis throughout the country, it is known that at least two points of care are involved: the Tertiary Reference Center (CRT) for tuberculosis and primary health care. These points of care have different but complementary technical competencies. The CRT is responsible for monitoring cases, prescribing medication, providing treatment, and requesting monitoring tests. Primary health care, on the other hand, is responsible for providing directly observed treatment, assessing contacts and referring people with suspected or confirmed resistance^2,4^.

On the other hand, there is a structural (and inevitable) fragmentation of CRT care, resulting in the dispersion of centers in the state, which can create care gaps and distance the professionals involved in care, increasing the risk of treatment interruption^7^.

Knowing the cartography of the care network for people with drug-resistant tuberculosis has the potential to visually and spatially represent the distribution of cases and health services, as well as identifying possible gaps in care coverage and areas most vulnerable to an increase in the burden of drug-resistant tuberculosis. The cartographic visualization of this information can support decision-making by health professionals and managers, as well as guiding the planning and targeting of actions aimed at eliminating tuberculosis as a public health problem.

The aim of this study was therefore to identify spatial clusters of risk and map the care network for people with drug-resistant tuberculosis in the state of São Paulo.

## METHODS

This is an ecological study, carried out by collecting secondary data from the Tuberculosis Special Treatment Information System (Site-TB). The state of São Paulo was chosen because it is one of the Federative Units with the highest number of people being treated for drug-resistant tuberculosis, as well as being the state with the second highest number of tuberculosis cases in the country^8^.

We used data from people who underwent treatment for drug-resistant tuberculosis in the state of São Paulo from 2013 to 2020, which was closed by July 2023. The interval chosen is justified by the year the system was implemented and the quality of the situation regarding the closure of drug-resistant tuberculosis cases.

Only validated cases were included, regardless of the type of resistance (primary or acquired/secondary) and the form of tuberculosis (pulmonary, extrapulmonary, or both). The following types of entry were considered: new cases, treatment interruption, failure, change of regimen, change in resistance pattern, recurrence, or no information on the type of entry on the case notification form. On the other hand, we excluded those whose case was closed due to a change in diagnosis, primary interruption of treatment, still being treated, transfer, as well as cases which did not provide information on the municipality of origin.

Access to the anonymized database occurred after a request to the Ministry of Health in August 2023. For sociodemographic characterization, the following variables were used: gender; race/color; schooling; age at the time of diagnosis of drug-resistant tuberculosis; initial resistance pattern and treatment outcome; and those relating to mapping (address of residence at the time of diagnosis and address of the CRT where treatment was carried out). It should be noted that “Treatment Success” was considered to be the sum of the cure and complete treatment outcomes.

Initially, the addresses were standardized. Next, the *Geocoding API* tool provided by the *Google Cloud* platform, which is characterized by cloud computing with modular management tools, was used to convert the addresses into geographic coordinate information (latitude and longitude). This process was carried out in R version 4.2.2.

For locations that were not geolocated using this procedure, the standardized addresses were searched for in *Google Earth Pro* version 7.3 and, based on the geographic coordinates located, a single vector shapefile was created for drug-resistant tuberculosis cases and CRTs.

Based on the point data, the Kernel density estimator was used for drug-resistant tuberculosis cases and CRT location. Conceptually, a surface is fitted over each point, which has a maximum value at the location of the point and decreases with its respective distance, reaching zero after a certain search radius. The density is then calculated by adding up the values of all the kernel surfaces where the points overlap^9^.

An important aspect of this analysis is the definition of the radius or band of influence of the estimator, since this parameter defines the smoothing of the Kernel surface. In this study, a fixed band was considered from the lowest value of the mean integrated squared error of the density estimator, calculated by cross-validation of least squares (LIK Selection)^10^. This procedure was carried out individually for each geolocated object and, for drug-resistant tuberculosis cases, the ideal radius of 10 km was estimated, and for CRT the band was 30 km.

The detection of spatial clusters of risk for drug-resistant tuberculosis was carried out using the purely spatial scan analysis proposed by Kulldorff and Nagarwalla^11^. The analysis establishes a flexible circular window on the map which is located at each of the centroids of the units of analysis, which in this case were the municipalities in the state of São Paulo.

This window of influence has a variable radius, from zero to a maximum limit, which in this study was defined as 10.0% of the state’s total population, according to the Gini index^12^. For each circular window, the alternative hypothesis is that there is a high/low risk inside the window compared to outside, and this calculation is carried out using the likelihood function for statistical testing purposes. In this sense, the “p” value is obtained using the Monte Carlo hypothesis test with 999 permutations.

The window with the maximum likelihood is the most probable cluster, i.e. the cluster with the lowest probability of having occurred at random. However, the analysis also identifies secondary clusters in the data set, depending on the cut-off value for rejecting the null hypothesis^13^.

Furthermore, considering the nature of counting the number of drug-resistant tuberculosis cases per municipality, the Poisson distribution model and the search for clusters with high and low risk were used simultaneously.

From the calculation of the number of cases observed and expected in each cluster, the relative risk (RR) is calculated, which is estimated from the risk in the cluster divided by the estimated risk outside the cluster. The risk, in turn, is a calculation of the number observed divided by the number expected within the cluster, divided by the number observed by the number expected outside the cluster^13^.

The scan analysis was carried out using the *SaTScan software* version 10.1.2 and the choropleth maps with the results of the spatial analysis were drawn up using the *QGIS* 3.34.2 tool. The significance level used in the study was 5%.

The project that gave rise to this article was approved by the Research Ethics Committee of the Ribeirão Preto School of Nursing, opinion number 3324619, in compliance with the ethical precepts established by Resolution 466/2012.

## RESULTS

From 2013 to 2020, 1,084 cases of drug-resistant tuberculosis were reported in the state of São Paulo. Most cases were male (n = 808; 74.5%), with a mean age of 39 years, median 37 years, and standard deviation of 13.9 years. As for race/color, most cases occurred in white (n = 517; 47.7%) and brown (n = 516; 47.6%) people (Table).

**Table:** Sociodemographic aspects of drug-resistant tuberculosis cases in the state of São Paulo, 2013 to 2020.

Variable	n	%
Sex		
Female	276	25.5
Male	808	74.5
Age group		
Up to 4 years old	1	0.1
5 to 9 years old	1	0.1
10 to 14 years old	4	0.4
15 to 19 years old	35	3.2
20 to 34 years old	425	39.2
35 to 49 years old	357	32.9
50 to 64 years old	217	20.0
65 to 79 years old	38	3.5
≥ 80 years old	6	0.6
Race/color		
Yellow	2	0.2
White	517	47.7
Indigenous	20	1.9
Black	157	14.5
Brown	359	33.1
Ignored/blank	25	2.3
No information	4	0.4
Schooling (in years)		
≥ 12	75	6.9
From 1 to 3	111	10.2
From 4 to 7	331	30.5
From 8 to 11	338	31.2
Ignored	165	15.2
None	28	2.6
No information	36	3.3

**Figure 1:**
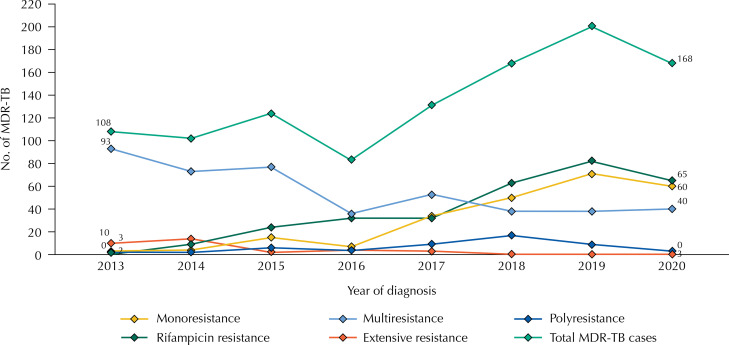
Number of new drug-resistant tuberculosis cases according to initial resistance pattern in São Paulo, 2013 to 2020.

From 2013 to 2020, the distribution of drug-resistant tuberculosis cases according to the initial resistance pattern was as follows: 448 cases (41.3%) of multidrug-resistant tuberculosis, 307 cases (28.3%) of rifampicin-resistant tuberculosis, and 33 cases (3.0%) of extensive resistance (Figure 1). In terms of treatment outcome, 684 cases (63.1%) were successfully treated; 233 cases (21.5%) discontinued treatment; 77 cases (7.1%) failed the regimen used; and 90 cases (8.3%) died, of which 61 (5.6%) were attributed to tuberculosis.

On average, 136 cases of drug-resistant tuberculosis were diagnosed annually, with 2019 recording the highest number of cases (n = 200) and 2016 the lowest (n = 83). The municipalities with the most cases were: São Paulo (n = 433), Ribeirão Preto (n 37), Santos (n = 37), Guarulhos (n = 31), and Campinas (n = 22), considering the individuals’ address of residence at the time of diagnosis.

**Figure 2:**
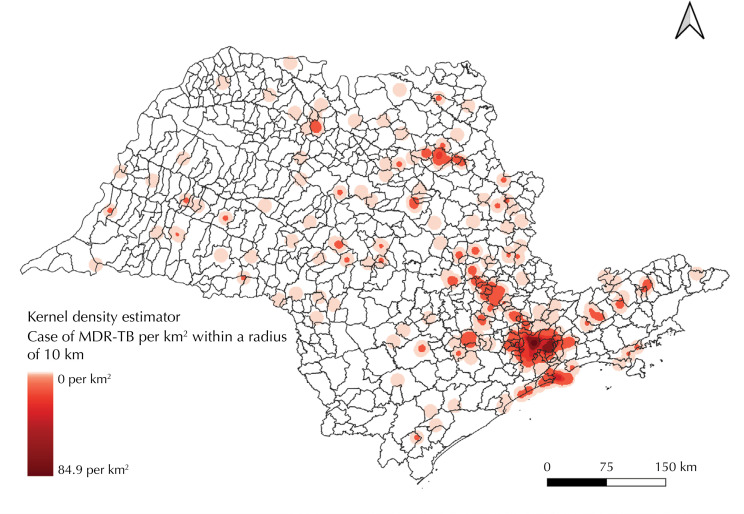
Kernel density estimates of geolocated drug-resistant tuberculosis cases. São Paulo, 2013 to 2020.

Geolocation showed 92.2% of the addresses of drug-resistant tuberculosis cases and 100% of the addresses of CRTs for treatment. The Kernel density estimator made it possible to identify a distribution pattern with specific variation between the different regions of the state of São Paulo. Drug-resistant tuberculosis cases were clustered close to the municipality of São Paulo, where the highest density of cases was observed, reaching 84.9 cases/km^2^. There was also a cluster of cases stretching from the state capital to the municipality of Campinas. In addition, there was a high density of cases in the Baixada Santista, located in the coastal region of the state, and in the municipality of Ribeirão Preto. Outside these areas, occasional cases have been identified in other regions, but without showing a pattern of spatial agglomeration, leaving large areas undiagnosed, mainly in the southeast and northwest (Figure 2).

**Figure 3:**
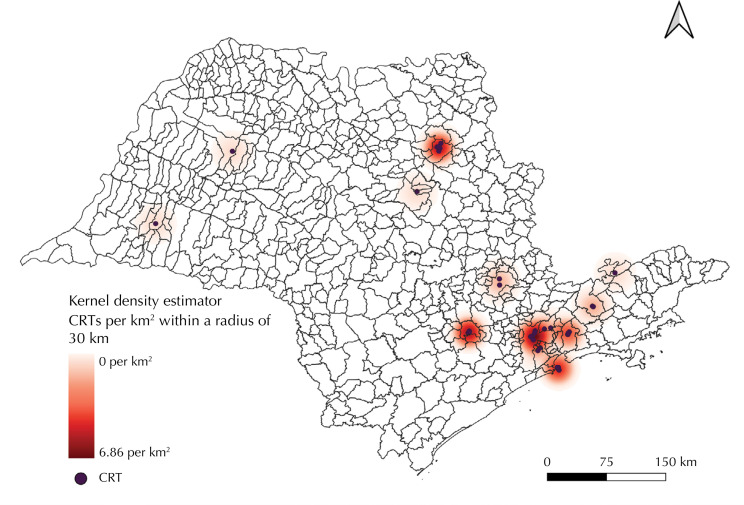
Kernel density estimates of tertiary referral centers for drug-resistant tuberculosis treatment. São Paulo, 2013 to 2020.

The CRTs for drug-resistant tuberculosis in the state also showed an important agglomeration pattern in the capital of São Paulo and in the municipalities of Sorocaba, Santos, and Ribeirão Preto, with up to 6.9 health units/km^2^. In the Kernel density estimator of CRTs, areas without care facilities for people with drug-resistant tuberculosis were observed, especially in the western, northwestern and southwestern portions of São Paulo. An example of the geographical absence of CRTs is revealed by the fact that, in a stretch of more than 500 km from the central region to the western edge of the state, there are only two health units available to care for this specific population (Figure 3).

CRT: Tertiary Reference Center

The two analyses show that the spatial pattern of CRTs was aligned with the distribution pattern of drug-resistant tuberculosis cases. The average straight-line distance between a drug-resistant tuberculosis case and a CRT in the municipalities of São Paulo, Ribeirão Preto, Santos, Guarulhos, and Campinas - which have the highest burden of the disease in the state - was 7.4 km (with a standard deviation of 5.7 km).

**Figure 4:**
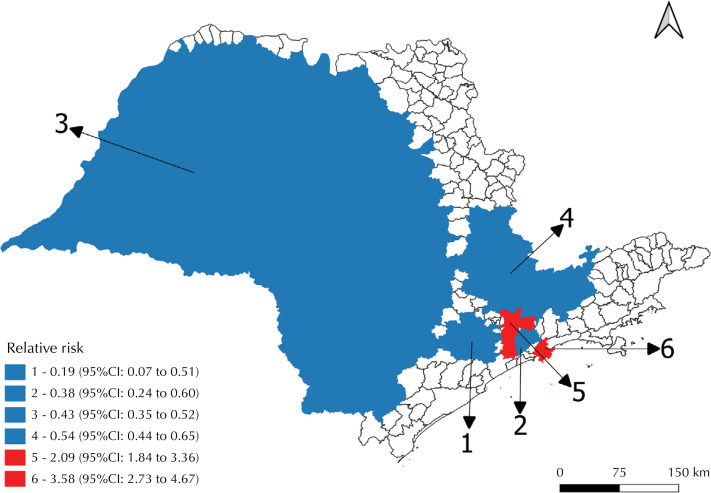
Distribution of clusters identified in the spatial scan analysis of drug-resistant tuberculosis cases. São Paulo, 2013 to 2020.

In contrast, in the other municipalities, the average distance to the nearest CRT was significantly greater, reaching 48 km (with a standard deviation of 43.3 km). In the spatial scan analysis of drug-resistant tuberculosis cases, six clusters were identified: four with a low risk of occurrence (1, 2, 3, and 4) and two with a high risk (5 and 6) (Figure 4).

Cluster 1 (RR = 0.19; 95%CI 0.07 to 0.51) covered 11 municipalities located to the west of the capital of São Paulo, with a population of 908,919 inhabitants and only four reported cases of drug-resistant tuberculosis, resulting in an incidence coefficient of 0.06 cases per 100,000 inhabitants. The scan analysis estimated an expected total of 20 cases in this region.

Cluster 2 (RR = 0.38; 95%CI 0.24 to 0.60) included five municipalities with 2,167,349 inhabitants and recorded 19 cases, resulting in an incidence coefficient of 0.1 cases per 100,000 inhabitants, compared to the expected 48 cases.

Cluster 3 (RR = 0.43; 95%CI 0.35 to 0.52) was the most extensive, with 417 municipalities, mainly in the central-western region of the state. This area, with 9,952,653 inhabitants, expected 221 cases of drug-resistant tuberculosis, while only 109 cases were reported, resulting in an incidence coefficient of 0.1 cases per 100,000 inhabitants.

Still in low-risk areas, cluster 4 (RR = 0.54; 95%CI 0.44 to 0.65) comprised 66 municipalities, with a population of 8,302,051 inhabitants and 109 observed cases (incidence coefficient of 0.2 cases per 100,000 inhabitants), in contrast to the 184 expected cases.

Among the risk areas for drug-resistant tuberculosis, cluster 5 (RR = 2.09; 95%CI 1.84 to 2.36), classified as the most likely (Most Likely Cluster), had 445 cases (incidence coefficient of 0.4 cases per 100,000 inhabitants), exceeding the 277 cases expected for the two municipalities that make up this area, with a total population of 12,474,050 inhabitants (São Paulo and Diadema).

Finally, cluster 6 (RR = 3.58; 95%CI 2.73 to 4.67) had the highest risk, reflected by the high RR value. This area included Santos and Guarujá, which together have 746,775 inhabitants and diagnosed 57 cases during the period analyzed (incidence coefficient of 1.0 case per 100,000 inhabitants), while the expectation was only 16 cases in this coastal region of the state.

## DISCUSSION

The study enabled the geospatial design of the occurrence of drug-resistant tuberculosis and treatment sites in the state of São Paulo, resulting in reliable mapping of the care network for people affected by resistant forms of tuberculosis.

The profile of the people identified in the sample studied is similar to that found in other studies on this subject, i.e. they are male, of white race/color, of economically active age^14-15^.

Globally, tuberculosis generally disproportionately affects marginalized and low-income people, and the racial profile can vary depending on the local context and the populations most affected. When it comes to drug-resistant tuberculosis, white people may have more access to health systems, which allows for a higher rate of detection and monitoring of cases of resistance. Conversely, other groups may be underdiagnosed or underreported^16^.

A predominance of multidrug-resistant forms was identified, followed by resistance to rifampicin. The implementation of MRT-TB has made it possible to diagnose tuberculosis more quickly and identify rifampicin resistance, which has contributed substantially to the increase in diagnosis with laboratory confirmation^17^and the identification of drug-resistant tuberculosis^18^.

The organization of health care in the state of São Paulo is divided into 17 Regional Health Care Networks (RRAS), whose definition was established considering the logic of organization through Health Care Networks^23^which, in turn, encompasses the installed care capacity to group primary health care and secondary care services, with the possibility of sharing tertiary care services between Health Regions^19^.

As far as the MRT-TB network is concerned, there is equipment in 25 of the 645 municipalities in São Paulo, although its use is growing^20^. Thus, as observed in this study, this technology is allocated to municipalities with a higher burden of drug-resistant tuberculosis, such as São Paulo, Campinas, and Ribeirão Preto, while other equipment is available in municipalities with a lower burden, such as Araçatuba, Bauru, and Presidente Prudente, covering all of the state’s RRAS^21^.

It is necessary to understand the strategies that the state of São Paulo uses to guarantee the testing of people from other municipalities that do not have MRT-TB, since the timely identification of cases is essential for breaking the chain of transmission of resistant strains, reducing mortality associated with drug-resistant tuberculosis and improving treatment outcomes^22^. Investing in the diagnosis of people with tuberculosis, especially the resistant forms, is essential for controlling the disease. However, this is still a major challenge for low- and middle-income countries such as Brazil^2^.

As for the treatment outcome, the success rate was higher than the national average, which is similar to other studies carried out in São Paulo. Arroyo et al.^23^, in their pre-pandemic study, showed that success rates have been progressive over the last few decades, which can be explained by the increased experience of specialized teams. However, negative outcomes, such as treatment interruption, are extremely worrying, as they further restrict the therapeutic possibilities of people with drug-resistant tuberculosis^24^.

CRTs are not present in all the state’s RRAS. In the analyses carried out, the strong clustering of CRTs and cases of drug-resistant tuberculosis stand out in the five municipalities in São Paulo with the highest burden of the disease: São Paulo, Ribeirão Preto, Santos, Guarulhos, and Campinas. This reveals, on the one hand, an agreement between the spatial pattern of resistant forms of tuberculosis and the provision of care and, on the other, questions about the difficulties encountered in identifying drug-resistant tuberculosis cases in regions that are more distant from specialized care.

The pattern of concentration of CRTs in São Paulo identified in the study reflects a scenario of agglomeration of specialized services in developed metropolitan and urban areas, such as São Paulo, Sorocaba, Santos, and Ribeirão Preto. This pattern is consistent with the literature on the distribution of health services in Brazil, which shows that the centralization of care units in large urban centers is the result of both greater personal demand and infrastructure^25^.

At the same time, peripheral areas, such as the west, northwest and southwest of the state, continue to lack adequate coverage, especially regarding the treatment of drug-resistant tuberculosis^26^. The absence of CRT in these regions highlights a problem that has already been addressed, namely that geographical inequality in the allocation of health resources tends to affect populations in rural or sparsely populated areas^27^.

A study^29^on the distribution of drug-resistant tuberculosis cases in Brazil also highlights the concentration of services in capital cities and more economically developed regions, while peripheral regions have less access to specialized units, which results in a greater risk of unfavorable outcomes for people who need treatment in these areas. This imbalance in access to treatment directly affects cure rates and the control of the disease, since the management of drug-resistant tuberculosis requires continuous and specialized follow-up, something that is scarce in more remote regions^28^.

Drug-resistant tuberculosis is a complex health condition that cannot be explained solely by the geographical distribution of CRTs, but by a set of individual variables (comorbidities and clinical presentation of the disease), as well as social inequalities and other barriers to access to health actions and services^7^.

Except for the municipality of São Paulo, the areas of greatest risk for drug-resistant tuberculosis did not occur in those with the highest burden of drug-resistant tuberculosis, such as the municipalities of Ribeirão Preto and Campinas. For these municipalities, there was a similarity between the cases observed and those expected for the period, showing that the care network in these municipalities is investigating the people under its responsibility in a timely manner.

It should also be noted that, in addition to São Paulo, the cities of Ribeirão Preto and Campinas stand out as centers with the largest number of devices for performing MRT-TB, facilitating the diagnosis of drug-resistant tuberculosis. These municipalities have a robust infrastructure for molecular diagnosis, which contributes to the early and efficient detection of cases of the disease^29^. The impact of this equipment has been analyzed in studies such as the one carried out in Ribeirão Preto, which showed an increase in case detection after the implementation of this technology^30^.

Given the risk situation in the municipalities of Diadema, Santos, and Guarujá, it is important to emphasize the need for these municipalities to create strategies for the timely diagnosis of drug-resistant tuberculosis cases, as well as for the appropriate treatment of cases.

There is an extensive low-risk area for drug-resistant tuberculosis in the center-west of the state, something that stands out due to the large number of municipalities. It is unclear whether this large territorial extension is really a protection area or a region with difficulties in detecting cases, which reinforces the need for future studies investigating the access of people living in this region to diagnosis of resistant forms of tuberculosis. On the other hand, clusters 1 and 2 are in places with a high concentration of CRTs, a fact that can be explained by the proximity of these clusters to the capital of São Paulo, whose municipalities may have at their disposal a larger contingent of qualified professionals to staff these services, as well as an adequate laboratory network.

Finally, the need for municipalities to comply with the cascade of care for people affected by drug-resistant tuberculosis is highlighted, as well as to identify mechanisms for overcoming care gaps, since these are essential for controlling the disease in São Paulo.

### Limitations and Strengths of the Study

As a limitation, we would highlight the possibility of underreporting drug-resistant tuberculosis cases, since we used secondary data from the Site-TB and this may have an impact on the results of this and other studies. It should be noted that no analyses were carried out identifying the interference of specific clinical aspects, such as differentiating between pulmonary or extrapulmonary forms or both, or the type of resistance, in this study.

The central strength of this research relates to the spatial identification of cases and specialized treatment units, making it possible to learn about disturbing aspects of drug-resistant tuberculosis care in the state of São Paulo and pointing to the need for further studies investigating the operational aspects involved in detecting and diagnosing cases in areas far from CRTs. We therefore recommend strengthening the infrastructure in the areas most at risk, expanding access to diagnosis and treatment, and training health professionals in the regions least served.

## CONCLUSIONS

This study has elucidated the dynamics of drug-resistant tuberculosis in the state of São Paulo, revealing spatial clusters that indicate areas of greater risk and, consequently, the need for targeted and strategic care. The mapping provides a detailed view of the distribution of cases and CRTs, highlighting the disproportion in access to treatment and the need to strengthen diagnostic strategies, especially in more vulnerable areas far from large urban centers. These findings indicate the need for health policies to expand diagnosis and treatment, improving the control of drug-resistant tuberculosis in the state of São Paulo.

Furthermore, the importance of mapping as a strategic tool for public health management is reinforced, enabling more targeted interventions and efficient allocation of resources. Future studies could deepen the analysis of the impact of the infrastructure of local services and identify additional factors that influence the outcomes of drug-resistant tuberculosis, with a view to more effective control of the disease and a more equitable health system.
