# Supplementary material for: Spatial clusters of risk and cartography of care for drug-resistant tuberculosis
Source: Rev Saude Publica. 2025 Jun 11;59:e11. doi: 10.11606/s1518-8787.2025059006489 (PMC12211794; doi:10.11606/s1518-8787.2025059006489)
Supplement: Translation

# Translation: Aglomerados espaciais de risco e cartografia da atenção à tuberculose drogarresistente

**Authors:** Jaqueline Garcia de Almeida Ballestero, José Nildo de Barros Silva, Luiz Henrique Arroyo, Daniele Maria Pelissari, Isabela Zaccaro Rigolin, Pedro Fredemir Palha, Aline Aparecida Monroe, Quézia Rosa Ferreira, Gilberto da Cruz Leal, Leonora de Oliveira Teixeira, Yury Bitencourt da Costa, Ione Carvalho Pinto, Rubia Laine de Paula Andrade, Ricardo Alexandre Arcêncio

**Affiliations:** I Brasil Universidade de São Paulo. Escola de Enfermagem de Ribeirão Preto. Ribeirão Preto, SP, Brasil; II Brasil Universidade de São Paulo. Faculdade de Saúde Pública. São Paulo, SP, Brasil

**Keywords:** Tuberculose, Tuberculose Resistente a Múltiplos Medicamentos, Mapeamento Geográfico, Sistemas de Saúde, Sistemas de Informação em Saúde, Enfermagem de Saúde Pública

## Abstract

**OBJETIVO:**

Identificar aglomerados espaciais de risco e cartografar a rede de cuidado às pessoas com tuberculose drogarresistente no estado de São Paulo.

**MÉTODOS:**

Trata-se de estudo do tipo ecológico, realizado por meio da coleta de dados provenientes do Sistema de Informação de Tratamentos Especiais de Tuberculose (Site-TB) de pessoas tratadas para tuberculose drogarresistente de 2013 a 2020, no estado de São Paulo. Foi realizada a cartografia por meio das técnicas Kernel e de estatística de varredura.

**RESULTADOS:**

Foram notificados 1.084 casos no período analisado. São Paulo, Ribeirão Preto, Santos, Guarulhos e Campinas foram os municípios que apresentaram o maior número de registros. O padrão espacial de aglomeração dos casos e dos Centros de Referência para o tratamento foram similares, com vazios de cobertura no sudoeste e noroeste do estado. Seis aglomerados espaciais foram identificados: quatro de baixo risco e dois de alto risco, localizados em São Paulo, Diadema, Santos e Guarujá.

**CONCLUSÕES:**

A concentração de casos e Centro de Referência Terciária em áreas metropolitanas evidencia desigualdades no acesso ao tratamento da tuberculose drogarresistente. Estes achados indicam a necessidade de políticas de saúde para expandir o diagnóstico e tratamento, melhorando o controle da tuberculose drogarresistente no estado de São Paulo.

## INTRODUÇÃO

A tuberculose drogarresistente consiste na resistência medicamentosa a um ou mais fármacos padronizados para o tratamento da tuberculose^1^. Em suas formas mais graves estão a tuberculose multirresistente e a tuberculose com resistência extensiva, ambas com alta possibilidade de gerar impactos negativos na vida das pessoas com a doença. A resistência pode ser detectada a partir do teste rápido molecular (TRM-TB), em casos que apresentam resistência à rifampicina^2-3^.

Os desafios relacionados ao diagnóstico das formas resistentes são complexos, perpassando por aspectos microbiológicos, clínicos, sociais, organizacionais e políticos^4^. Em 2022, dos 410.000 casos de tuberculose multirresistente ou resistência à rifampicina estimados pela Organização Mundial de Saúde (OMS), apenas 175.650 (42,8%) foram diagnosticados e tiveram o tratamento iniciado^4^. No Brasil, foram notificados 1.104 casos de tuberculose drogarresistente no mesmo ano, o que equivale a cerca de 67,1% do total estimado pela OMS para o país^1^. E, ainda, o estado de São Paulo teve a segunda maior proporção de casos de tuberculose drogarresistente, sendo o município de São Paulo o terceiro em número de casos no contexto brasileiro, de 2019 a 2023^5^.

Assim, apesar do Brasil não estar incluído na lista dos 30 países com maior carga de tuberculose drogarresistente, o perfil epidemiológico da tuberculose no território nacional leva a OMS a considerar o País como de alto risco para disseminação de formas resistentes^6^, o que representa uma considerável questão de saúde pública.

Em se tratando da organização da atenção à tuberculose drogarresistente em todo o país, sabe-se que pelo menos dois pontos assistenciais estão envolvidos: o Centro de Referência Terciária (CRT) à tuberculose e a atenção primária à saúde. Esses pontos de atenção possuem competências técnicas diferentes, mas complementares. O CRT é responsável pelo acompanhamento dos casos, pela prescrição de medicamentos, pelo fornecimento de tratamentos e pela solicitação de exames de monitoramento. Já a atenção primária à saúde é encarregada de realizar o tratamento diretamente observado, a avaliação dos contatos, bem como o encaminhamento das pessoas que apresentam suspeita ou confirmação de resistência^2,4^.

Observa-se, em contrapartida, uma fragmentação estrutural (e inevitável) da atenção dos CRT, resultando na dispersão dos centros no estado, o que pode gerar vazios assistenciais e distanciar os profissionais envolvidos com a assistência, aumentando o risco da interrupção do tratamento^7^.

Conhecer a cartografia da rede de cuidado à pessoa com tuberculose drogarresistente tem o potencial de representar, de forma visual e espacial, a distribuição dos casos e dos serviços de saúde, além de identificar possíveis lacunas na cobertura assistencial e áreas mais vulneráveis ao aumento da carga de tuberculose drogarresistente. A visualização cartográfica destas informações pode apoiar a tomada de decisões por parte dos profissionais de saúde e gestores, além de orientar o planejamento e o direcionamento de ações voltadas para a eliminação da tuberculose como problema de saúde pública.

Assim, o presente estudo teve por objetivo identificar aglomerados espaciais de risco e cartografar a rede de cuidado às pessoas com tuberculose drogarresistente no estado de São Paulo.

## MÉTODOS

Trata-se de estudo do tipo ecológico, realizado por meio da coleta de dados secundários provenientes do Sistema de Informação de Tratamentos Especiais em Tuberculose (Site-TB). O estado de São Paulo foi escolhido por ser uma das Unidades Federativas que apresentam maior quantidade de pessoas em tratamento da tuberculose drogarresistente, além de ser o segundo estado com mais casos de tuberculose no país^8^.

Utilizou-se dados de pessoas que realizaram o tratamento de tuberculose drogarresistente no estado de São Paulo de 2013 a 2020, cujo encerramento ocorreu até julho de 2023. O intervalo escolhido justifica-se pelo ano de implementação do sistema e pela qualidade da situação de encerramento dos casos de tuberculose drogarresistente.

Incluiu-se apenas os casos validados, independente do tipo de resistência (primária ou adquirida/secundária) e da forma de tuberculose (pulmonar, extrapulmonar ou ambas). Considerou-se os seguintes tipos de entrada: casos novos, interrupção do tratamento, falência, mudança de esquema, mudança do padrão de resistência, recidiva ou sem informação do tipo de entrada na ficha de notificação do caso. Excluiu-se, em contrapartida, aqueles que tiveram o caso encerrado por mudança no diagnóstico, interrupção primária do tratamento, ainda estar em tratamento, transferência, bem como os casos que não apresentaram informações quanto ao município de procedência.

O acesso ao banco de dados anonimizado ocorreu após solicitação ao Ministério da Saúde em agosto de 2023. Para a caracterização sociodemográfica utilizou-se as variáveis: sexo; raça/cor; escolaridade; idade no momento do diagnóstico da tuberculose drogarresistente; padrão de resistência inicial e desfecho do tratamento; e as referentes ao mapeamento (endereço de residência no momento do diagnóstico e endereço do CRT no qual realizou tratamento). Destaca-se que foi considerada como “Sucesso de Tratamento” a soma entre os desfechos de cura e de tratamento completo.

Inicialmente, os endereços foram padronizados. Em seguida, foi utilizada a ferramenta *API Geocoding* disponibilizada pela plataforma *Google Cloud*, que se caracteriza pela computação em nuvem com ferramentas de gerenciamento modulares, no objetivo de converter os endereços em informações de coordenadas geográficas (latitude e longitude). Esse processo foi conduzido no R versão 4.2.2.

Para as localidades não geolocalizadas por meio desse procedimento, realizou-se a busca dos endereços padronizados no *software Google Earth Pro* versão 7.3 e, a partir das coordenadas geográficas localizadas, foi elaborado um único arquivo vetorial *shapefile* para os casos de tuberculose drogarresistente e para os CRT.

A partir dos dados pontuais, foi realizado o estimador de densidade de Kernel para os casos de tuberculose drogarresistente e localidade do CRT. Conceitualmente, uma superfície é ajustada sobre cada ponto, que apresenta valor máximo na localização do ponto e diminui com o seu respectivo distanciamento, alcançando o valor zero após um determinado raio de busca. Assim, a densidade é calculada adicionando os valores de todas as superfícies do Kernel onde se sobrepõem os pontos^9^.

Um importante aspecto desta análise é a definição do raio ou banda de influência do estimador, haja vista que este parâmetro define o alisamento da superfície de Kernel. Neste estudo, foi considerada uma banda fixa a partir do menor valor do erro quadrático integrado médio do estimador de densidade, calculado pela validação cruzada de mínimos quadrados (Seleção LIK)^10^. Esse procedimento foi realizado individualmente para cada objeto geolocalizado e, para os casos de tuberculose drogarresistente, foi estimado o raio ideal de 10 km e, para os CRT, a banda foi de 30 km.

A detecção dos aglomerados espaciais de risco para tuberculose drogarresistente foi conduzida a partir da Análise de Varredura puramente espacial proposta por Kulldorff e Nagarwalla^11^. A análise estabelece uma janela circular flexível no mapa que está localizada em cada um dos centroides das unidades de análise, que, no referido caso, foram os municípios do estado de São Paulo.

Esta janela de influência possui raio variável, saindo do zero até um limite máximo, que neste estudo foi definido como 10,0% da população total do estado, conforme índice de Gini^12^. Para cada janela circular, a hipótese alternativa é de que existe um risco elevado/baixo no interior da janela em comparação ao exterior, e este cálculo é conduzido pela função de verossimilhança com a finalidade de teste estatístico. Nesse sentido, o valor de “p” é obtido por meio do teste de hipótese de Monte Carlo com 999 permutações.

A janela com a máxima verossimilhança é o aglomerado mais provável, ou seja, o aglomerado que tem a menor probabilidade de ter ocorrido ao acaso. Entretanto, a análise também identifica aglomerados secundários no conjunto de dados, a depender do valor de corte para rejeitar a hipótese nula^13^.

Ademais, ao considerar a natureza de contagem do número de casos de tuberculose drogarresistente por município, foram utilizados o modelo de distribuição de Poisson e a busca por aglomerados com risco alto e baixo simultaneamente.

A partir do cálculo do número de casos observados e esperados em cada aglomerado, é calculado o risco relativo (RR), ao qual é estimado a partir do risco no aglomerado dividido pelo risco estimado fora do aglomerado. O risco, por sua vez, é um cálculo entre o número observado dividido pelo esperado dentro do aglomerado, dividido pelo observado pelo esperado fora do aglomerado^13^.

A análise de varredura foi realizada com auxílio do *software SaTScan* versão 10.1.2 e os mapas coropléticos com os resultados das análises espaciais foram elaborados com ajuda da ferramenta *QGIS* 3.34.2. O nível de significância utilizado no estudo foi de 5%.

O projeto que deu origem ao presente artigo foi aprovado pelo Comitê de Ética em Pesquisa da Escola de Enfermagem de Ribeirão Preto, parecer número 3324619, atendendo aos preceitos éticos estabelecidos pela Resolução nº 466/2012.

## RESULTADOS

De 2013 a 2020, foram notificados 1.084 casos de tuberculose drogarresistente no estado de São Paulo. A maioria dos casos era do sexo masculino (n = 808; 74,5%), com idade média de 39 anos, mediana 37 anos e desvio-padrão de 13,9 anos. Quanto à raça/cor, a maioria dos casos ocorreu em pessoas brancas (n = 517; 47,7%) e pardas (n = 516; 47,6%) (Tabela).

**Tabela:** Aspectos sociodemográficos dos casos de tuberculose drogarresistente no estado de São Paulo, 2013 a 2020.

Variável	n	%
Sexo		
Feminino	276	25,5
Masculino	808	74,5
Faixa etária		
Até 4 anos	1	0,1
5 a 9 anos	1	0,1
10 a 14 anos	4	0,4
15 a 19 anos	35	3,2
20 a 34 anos	425	39,2
35 a 49 anos	357	32,9
50 a 64 anos	217	20,0
65 a 79 anos	38	3,5
≥ 80 anos	6	0,6
Raça/cor		
Amarela	2	0,2
Branca	517	47,7
Indígena	20	1,9
Preta	157	14,5
Parda	359	33,1
Ignorado/em branco	25	2,3
Sem informação	4	0,4
Escolaridade (em anos)		
≥ 12	75	6,9
De 1 a 3	111	10,2
De 4 a 7	331	30,5
De 8 a 11	338	31,2
Ignorada	165	15,2
Nenhuma	28	2,6
Sem informação	36	3,3

De 2013 a 2020, a distribuição dos casos de tuberculose drogarresistente segundo o padrão de resistência inicial foi a seguinte: 448 casos (41,3%) de tuberculose multirresistente, 307 casos (28,3%) de tuberculose com resistência à rifampicina e 33 casos (3,0%) de tuberculose extensiva (Figura 1). Quanto ao desfecho de tratamento, 684 casos (63,1%) tiveram sucesso no tratamento; 233 casos (21,5%), interromperam o tratamento; 77 casos (7,1%), apresentaram falência ao esquema utilizado; e 90 casos (8,3%) evoluíram para óbito, dos quais 61 (5,6%) foram atribuídos à tuberculose.

**Figura 1:** Número de casos novos de tuberculose drogarresistente segundo o padrão de resistência inicial em São Paulo, 2013 a 2020.

Em média, foram diagnosticados anualmente 136 casos de tuberculose drogarresistente, com 2019 registrando o maior número de casos (n = 200) e 2016 o menor (n = 83). Os municípios com mais casos foram: São Paulo (n = 433), Ribeirão Preto (n = 37), Santos (n = 37), Guarulhos (n = 31) e Campinas (n = 22), considerando o endereço de residência dos indivíduos no momento do diagnóstico.

A geolocalização mostrou 92,2% dos endereços dos casos de tuberculose drogarresistente e 100% dos endereços dos CRT para o tratamento. O estimador de densidade de Kernel permitiu identificar um padrão de distribuição com variação específica entre as diferentes regiões do estado de São Paulo. Os casos de tuberculose drogarresistente estavam aglomerados próximos ao município de São Paulo, onde foi observada maior densidade de casos, atingindo 84,9 casos/km^2^. Observou-se também uma aglomeração de casos que se estende da região da capital do estado até o município de Campinas. Além disso, destaca-se alta densidade de casos na baixada santista, localizada na região litorânea do estado, e no município de Ribeirão Preto. Fora dessas áreas, foram identificados casos pontuais em outras regiões, mas sem evidenciar um padrão de aglomeração espacial, deixando grandes áreas sem diagnósticos, principalmente nas áreas sudeste e noroeste (Figura 2).

**Figura 2:** Estimativas de densidade de Kernel dos casos geolocalizados de tuberculose drogarresistente. São Paulo, 2013 a 2020.

Os CRT para tuberculose drogarresistente no estado mostraram também um importante padrão de aglomeração na capital paulista e nos municípios de Sorocaba, Santos e Ribeirão Preto, com até 6,9 unidades de saúde/km^2^. No estimador de densidade de Kernel dos CRT, foram observadas áreas sem unidades de atendimento para pessoas com tuberculose drogarresistente, especialmente nas porções oeste, noroeste e sudoeste de São Paulo. Um exemplo da ausência geográfica de CRT é revelado pelo fato de que, em uma extensão de mais de 500 km da região central até o limite oeste do estado, existem apenas duas unidades de saúde disponíveis para atender essa população específica (Figura 3).

**Figura 3:** Estimativas de densidade de Kernel dos Centros de Referência Terciária para tratamento da tuberculose drogarresistente. São Paulo, 2013 a 2020.

CRT: Centro de Referência Terciária

As duas análises permitem observar que o padrão espacial dos CRT estava alinhado com o padrão de distribuição dos casos de tuberculose drogarresistente. A distância média em linha reta entre um caso de tuberculose drogarresistente e um CRT nos municípios de São Paulo, Ribeirão Preto, Santos, Guarulhos e Campinas – que apresentam a maior carga da doença no estado – foi de 7,4 km (com desvio-padrão de 5,7 km).

Em contraste, nos demais municípios, a distância média até o CRT mais próximo do domicílio foi significantemente maior, alcançando 48 km (com desvio-padrão de 43,3 km). Na análise de varredura espacial dos casos de tuberculose drogarresistente, foram identificados seis aglomerados: quatro com baixo risco de ocorrência (1, 2, 3 e 4) e dois com alto risco (5 e 6) (Figura 4).

**Figura 4:** Distribuição dos aglomerados identificados na análise da varredura espacial dos casos de tuberculose drogarresistente. São Paulo, 2013 a 2020.

O aglomerado 1 (RR = 0,19; IC95% 0,07 a 0,51) abrangeu 11 municípios localizados no oeste da capital paulista, com população de 908.919 habitantes e apenas quatro casos de tuberculose drogarresistente notificados, resultando em um coeficiente de incidência de 0,06 casos por 100 mil habitantes. A análise de varredura estimou um total esperado de 20 casos nesta região.

O aglomerado 2 (RR = 0,38; IC95% 0,24 a 0,60) incluiu cinco municípios com 2.167.349 habitantes e registrados 19 casos, resultando em um coeficiente de incidência de 0,1 casos por 100 mil habitantes, em comparação com o esperado de 48 casos.

O aglomerado 3 (RR = 0,43; IC95% 0,35 a 0,52) foi o mais extenso, com 417 municípios, principalmente na região centro-oeste do estado. Essa área, com 9.952.653 habitantes, tinha expectativa de 221 casos de tuberculose drogarresistente, enquanto foram notificados apenas 109 casos, resultando em um coeficiente de incidência de 0,1 casos por 100 mil habitantes.

Ainda nas áreas de baixo risco, o aglomerado 4 (RR = 0,54; IC95% 0,44 a 0,65) compreendeu 66 municípios, com população de 8.302.051 habitantes e 109 casos observados (coeficiente de incidência de 0, 2 casos por 100 mil habitantes), em contraste com os 184 casos esperados.

Entre as áreas de risco para tuberculose drogarresistente, o aglomerado 5 (RR = 2,09; IC95% 1,84 a 2,36), classificado como o mais provável (*Most Likely Cluster*), apresentou 445 casos (coeficiente de incidência de 0,4 casos por 100 mil habitantes), superando os 277 casos esperados para os dois municípios que compõem essa área, com população total de 12.474.050 habitantes (São Paulo e Diadema).

Por fim, o aglomerado 6 (RR = 3,58; IC95% 2,73 a 4,67) apresentou o maior risco, refletido pelo elevado valor de RR. Esta área abrangeu Santos e Guarujá, que juntos possuem 746.775 habitantes e diagnosticaram 57 casos durante o período analisado (coeficiente de incidência de 1,0 caso por 100 mil habitantes), enquanto a expectativa era de apenas 16 casos nesta região litorânea do estado.

## DISCUSSÃO

O estudo permitiu o desenho geoespacial da ocorrência da tuberculose drogarresistente e dos locais de tratamento no estado de São Paulo, resultando na cartografia da rede de cuidado às pessoas acometidas pelas formas resistentes de tuberculose de forma confiável.

O perfil das pessoas identificado na amostra estudada se assemelha com aquele encontrado em outras investigações sobre essa temática, isto é, são pessoas do sexo masculino, de raça/cor branca, com idade economicamente ativa^14-15^.

Em nível global, a tuberculose geralmente afeta de maneira desproporcional pessoas marginalizadas e de baixa renda, e o perfil racial pode variar dependendo do contexto local e das populações mais afetadas. Em se tratando da tuberculose drogarresistente, pessoas brancas podem ter mais acesso a sistemas de saúde, o que permite maior taxa de detecção e monitoramento dos casos de resistência. De modo oposto, outros grupos podem ser subdiagnosticados ou subnotificados^16^.

Foi identificada a predominância das formas multirresistentes, seguida pela resistência à rifampicina. A implantação do TRM-TB tem possibilitado um diagnóstico mais rápido da tuberculose e a identificação da resistência à rifampicina, o que vem contribuindo substancialmente para o aumento do diagnóstico com confirmação laboratorial^17^ e a identificação da tuberculose drogarresistente^18^.

A organização da assistência à saúde no estado de São Paulo se dá por meio da divisão em 17 Redes Regionais de Atenção à Saúde (RRAS), cuja definição foi estabelecida considerando a lógica de organização por meio de Redes de Atenção à Saúde^23^ que, por sua vez, abarca a capacidade assistencial instalada de forma a agrupar serviços de atenção primária à saúde e da atenção secundária, sendo admitida a possibilidade de compartilhamento de serviços de atenção terciária entre Regiões de Saúde^19^.

No que diz respeito à rede de TRM-TB, há a presença de equipamentos em 25 dos 645 municípios paulistas, embora a sua utilização esteja em crescente expansão^20^. Assim, como observado neste estudo, há alocação desta tecnologia em municípios com maior carga da tuberculose drogarresistente como São Paulo, Campinas, Ribeirão Preto, sendo outros equipamentos disponíveis em municípios com menor carga, como Araçatuba, Bauru e Presidente Prudente, contemplando todas as RRAS do estado^21^.

É necessário entender as estratégias que o estado paulista utiliza com vistas à garantia de testagem de pessoas dos outros municípios que não dispõem do TRM-TB, uma vez que a identificação oportuna dos casos é essencial para a quebra da cadeia de transmissão das cepas resistentes, redução da mortalidade associada à tuberculose drogarresistente e melhora dos desfechos de tratamento^22^. Investir no diagnóstico de pessoas com tuberculose, especialmente nas formas resistentes, é fundamental para o controle da doença. No entanto, isso ainda representa um grande desafio para países de baixa e média renda, como o Brasil^2^.

Quanto ao desfecho do tratamento, foi observada uma taxa de sucesso superior à média nacional, dados que se assemelham a outras pesquisas realizadas no cenário paulista. Arroyo et al.^23^, em seu estudo pré-pandemia, evidenciaram que as taxas de sucesso foram progressivas ao longo das últimas décadas, o que pode ser justificado pela ampliação da experiência das equipes especializadas. No entanto, os desfechos negativos, como a interrupção do tratamento, são extremamente preocupantes, pois restringem ainda mais as possibilidades terapêuticas das pessoas com tuberculose drogarresistente^24^.

Os CRT não estão presentes em todas as RRAS do Estado. Nas análises realizadas, destacam-se a forte aglomeração de CRT e os casos de tuberculose drogarresistente nos cinco municípios paulistas com maior carga da doença: São Paulo, Ribeirão Preto, Santos, Guarulhos e Campinas. Revela-se, por um lado, sintonia entre o padrão espacial das formas resistentes de tuberculose e a oferta da atenção e, por outro, questionamentos acerca das dificuldades encontradas para a identificação dos casos de tuberculose drogarresistente em regiões mais distantes do cuidado especializado.

O padrão de concentração dos CRT em São Paulo, identificado no estudo, reflete um cenário de aglomeração de serviços especializados em áreas metropolitanas e urbanas desenvolvidas, como São Paulo, Sorocaba, Santos e Ribeirão Preto. Esse padrão é consistente com a literatura sobre a distribuição de serviços de saúde no Brasil, que mostra que a centralização de unidades de atendimento em grandes centros urbanos é resultado tanto de maior demanda pessoal quanto de infraestrutura^25^.

Ao mesmo passo, áreas periféricas, como o oeste, noroeste e sudoeste do estado, continuam carentes de cobertura adequada, especialmente no que diz respeito ao tratamento de tuberculose drogarresistente^26^. A ausência de CRT nessas regiões evidencia um problema já abordado antes, isto é, a desigualdade geográfica na alocação de recursos de saúde tende a afetar populações em áreas rurais ou de baixa densidade populacional^27^.

Um estudo^29^ sobre a distribuição de casos de tuberculose drogarresistente no Brasil também destaca a concentração de serviços em capitais e regiões economicamente mais desenvolvidas, enquanto regiões periféricas apresentam menor acesso a unidades especializadas, o que resulta em maior risco de desfechos desfavoráveis para pessoas que necessitam de tratamento nessas áreas. Esse desequilíbrio no acesso ao tratamento afeta diretamente as taxas de cura e o controle da doença, uma vez que a gestão da tuberculose drogarresistente requer acompanhamento contínuo e especializado, algo escasso em regiões mais afastadas^28^.

A tuberculose drogarresistente é uma condição de saúde complexa que não pode ser explicada somente pela distribuição geográfica dos CRT, mas pelo conjunto de variáveis individuais (comorbidades e apresentação clínica da doença), bem como pelas desigualdades sociais e outras barreiras de acesso às ações e aos serviços de saúde^7^.

Exceto o município de São Paulo, as áreas de maior risco para tuberculose drogarresistente não ocorreram naquelas com maior carga de tuberculose drogarresistente, como os municípios de Ribeirão Preto e Campinas. Para estes, houve similaridade entre os casos observados e os esperados para o período, mostrando que a rede de atenção destes municípios está investigando de forma oportuna as pessoas sob sua responsabilidade.

Cabe destacar ainda que, além de São Paulo, as cidades de Ribeirão Preto e Campinas se destacam como centros com maior número de equipamentos para realização do TRM-TB, facilitando o diagnóstico da tuberculose drogarresistente. Esses municípios possuem infraestrutura robusta para o diagnóstico molecular, o que contribui para a detecção precoce e eficiente de casos da doença^29^. O impacto desse equipamento foi analisado em estudos como o realizado em Ribeirão Preto, que mostrou aumento na detecção dos casos após a implementação dessa tecnologia^30^.

Perante a situação de risco dos municípios de Diadema, Santos e Guarujá, há que se destacar a necessidade de esses municípios criarem estratégias para o diagnóstico oportuno dos casos de tuberculose drogarresistente, bem como para o tratamento adequado dos casos.

Existe uma extensa área de baixo risco para a tuberculose drogarresistente no centro-oeste do estado, algo que se destaca pela grande quantidade de municípios. Não está claro se essa grande extensão territorial é realmente uma área de proteção ou uma região com dificuldades para detecção dos casos, o que reforça a necessidade de estudos futuros que investiguem o acesso das pessoas que residem nesta região ao diagnóstico das formas resistentes de tuberculose. Em contrapartida, os aglomerados 1 e 2 encontram-se em locais com alta concentração de CRT, fato que pode ser explicado pela proximidade de tais aglomerados em relação à capital paulista, cujos municípios podem ter ao seu dispor maior contingente de profissionais qualificados para compor os referidos serviços, bem como adequada rede laboratorial.

Por fim, destaca-se a necessidade de os municípios cumprirem com a cascata de cuidado à pessoa acometida pela tuberculose drogarresistente, bem como identificar mecanismos de superação de vazios assistenciais, uma vez que são imprescindíveis para o controle da doença no contexto paulista.

### Limitação e fortalezas do estudo

Como limitação, destaca-se a possibilidade de subnotificação de casos de tuberculose drogarresistente, uma vez que foram utilizados dados secundários provenientes do Site-TB e que isso pode impactar os resultados deste e de outros estudos. Há de se destacar que não foram realizadas análises identificando a interferência de aspectos clínicos específicos, como a diferenciação entre as formas pulmonares, extrapulmonares ou ambas, bem como o tipo de resistência, no presente estudo.

A fortaleza central desta pesquisa se relaciona à identificação espacial dos casos e as unidades de tratamento especializado, possibilitando conhecer aspectos inquietantes da atenção à tuberculose drogarresistente no estado de São Paulo e apontando para a necessidade de outros estudos que investiguem os aspectos operacionais envolvidos na detecção e diagnóstico de casos em áreas distantes dos CRT. Desta forma, recomenda-se fortalecer a infraestrutura nas áreas de maior risco, ampliar o acesso ao diagnóstico e tratamento, e capacitar profissionais de saúde nas regiões menos assistidas.

## CONCLUSÕES

O presente estudo elucidou a dinâmica da tuberculose drogarresistente no estado de São Paulo, revelando aglomerados espaciais que indicam áreas de maior risco e, consequentemente, a necessidade de uma atenção direcionada e estratégica. A cartografia elaborada proporciona uma visão detalhada da distribuição dos casos e dos CRT, destacando a desproporção no acesso ao tratamento e a necessidade de fortalecer as estratégias de diagnóstico, especialmente em áreas mais vulneráveis e afastadas dos grandes centros urbanos. Estes achados indicam a necessidade de políticas de saúde para expandir o diagnóstico e tratamento, melhorando o controle da tuberculose drogarresistente no estado de São Paulo.

Ademais, reforça-se a importância da cartografia como ferramenta estratégica para a gestão da saúde pública, possibilitando intervenções mais direcionadas e alocação eficiente de recursos. Futuros estudos podem aprofundar a análise sobre o impacto da infraestrutura dos serviços locais e identificar fatores adicionais que influenciam os desfechos da tuberculose drogarresistente, visando um controle mais eficaz da doença e um sistema de saúde mais equitativo.
